# Caste Exclusion and Health Discrimination in South Asia: A Systematic Review

**DOI:** 10.1177/10105395211014648

**Published:** 2021-05-24

**Authors:** Raksha Thapa, Edwin van Teijlingen, Pramod Raj Regmi, Vanessa Heaslip

**Affiliations:** 1 Bournemouth University, Bournemouth, UK; 2 Manmohan Memorial Institute of Health Sciences, Kathmandu, Nepal; 3 Datta Meghe Institute of Medical Sciences, Wardha, India; 4 University of Stavanger, Stavanger, Norway

**Keywords:** Asia, caste, Dalits, social exclusion, health equity, health access, health utilization

## Abstract

The caste system is social stratification system that has been used over the last 3 millennia. This review aims to investigate caste-based inequity in health care utilization in South Asia, particularly focusing those at the bottom of the caste hierarchy, commonly known as Dalit communities. A systematic methodology was followed; key databases (including CINAHL, Medline, SocINDEX, PubMed, Nepjol, JSTOR, and ASSIA) were searched for relevant articles published before October 2019, using comprehensive search strategy in accordance with the PRISMA guidelines. In total 15,109 papers were found, and from these, nine selected papers were included in the review. The papers focused on studies in both India (n = 7) and Nepal (n = 2) and utilized a range of methods including qualitative (n = 2), quantitative (n = 3), and mixed methods (n = 4) approaches. The review identified four main themes: stigma, poverty, cultures and beliefs, and health care. Caste-based inequity impacts upon all aspects of an individual’s well-being including violence and everyday life risks. Caste also impacts upon individuals’ opportunities to access education, employment, and health care. Dalits appear to experience this more significantly due to both poverty and their caste status, which increases their vulnerability to health risks.

## What We Already Know

- Health inequity is determined by broader social factors such as socioeconomic status, education, poverty, gender, and caste.
- In Nepal and India, Dalits are the most marginalized people at the bottom of social hierarchy who experience most barriers to accessing public services.
- Social stratification by caste is made worse by discrimination and a lack of education.

## What This Article Adds

- Understanding experiences and challenges associated to health service uptake of Dalit minorities due to caste discrimination.
- Identifying gaps between caste and health equity and highlights possible factors underlying discrimination.
- Aspects that limit access to health services among Dalit communities, including stigma, poverty, culture and beliefs, and health care.

## Introduction

Discrimination impacts upon wider determinants of health such as education, employment, income, and housing.^1^ Caste is a fundamental determinant of social exclusion and development; indeed, international human rights organizations argue that worldwide more than 260 million individuals experience this exclusion.^2^ To understand caste discrimination, it is important to clarify the caste system and those groups most disadvantaged by it, generally called “lower caste,” and widely known as “Dalits.”^3^

The 3000-year-old caste system is one of the oldest social hierarchies and it forms the foundation of Hindu society.^4^ This system includes four divisions: “Brahmins,” priests; “Kshetriyas,” warriors; “Vaishyas,” merchants; and “Sudras,” servants. Underneath these castes lies “Ati-Sudra,” Dalits, also known as untouchables.^5^ Dalits rest at the bottom of the caste hierarchy and are often perceived as water polluting, untouchables, impure, doom, pariganit, and tallo jat.^6^ Dalits are an outcasted group, often referred to as “Broken men” in Hindu scriptures; Ambedkar noted that they are referred to as “Protestant Hindus” or “Harijans” or “untouchables and depressed class” or “scheduled caste.”^7^ However, the word “Dalit” is widely recognized, and the Dalit movement emphasizes the importance of accepting this term to note the continuous discrimination based on caste.^5^ Caste discrimination is a highly sensitive and politicized issue in Asia,^3,6,8^ especially India and Nepal where more than 80% of the population are Hindu.^9^ Criticizing the caste system is hindered by a fear of offending religious or cultural sensitivities among Hindus.^8^

Studies have reported that people from high castes experience freedom and high status, whereas people from lower caste are restricted from attending schools, temples, and courthouses. Furthermore, they are restricted in trading goods, labor, and were stigmatized through the practice of untouchability.^10^ Caste-based inequity also impacts upon employment limiting Dalits to low-status occupations such as making brooms, baskets, ropes, sex workers, and domestic labors.^11^ Dalits are also linked to occupations such as scavengers, sweepers, rag pickers, and coolies, which are considered to be dirty, unimportant, and unhygienic and hence associated with religious notions of purity-pollution.^12^ Dalits have also been prevented from establishing equal relationships in social, educational, political, and economic domains in comparison to those from higher caste.^13^ The Dalits are especially vulnerable and isolated due to this notion of untouchability in the caste system.^14^

Hinduism is a very patriarchal religion, and in Hindu-dominated societies women’s status is traditionally lower than that of men.^15^ Caste-based disparities interact with patriarchy and together play a significant role in further isolating *Dalit women* who are also known as the “Dalit among Dalits.” As such, Dalit women experience double discrimination based upon their caste identity and their gender. They are largely ignored and experience discrimination leading to health inequity, especially with regard to maternal health services.^16^ Caste and gender often render Dalit women and girls, particularly vulnerable to being excluded from schooling, generating literacy barriers.^17^ In Nepal, there is a paucity of studies on the health perspective of women, especially Dalit and disabled women accessing and using maternal health services.^18^ Due to social and religious practices, women from these communities are more vulnerable to sexual exploitation. Similarly, due to caste discrimination Dalit women experience difficulties accessing social, economic, civil rights, and entitlement. The main challenges they face include the following: poor health, reduced education, economic deprivation due to limited employment opportunities, reduced public service and political participation, violence and atrocities, prostitution, and gender inequality.^19^ In this review, we seek to also understand gender aspects of caste by presenting challenges Dalit women face.

A large number of Dalits in rural areas in India are deprived from or are refused access to health services due to their social status.^20^ The state of Nepal aimed to address the issues of caste discrimination by developing affirmative regulation and policies (health policies, nutrition health policy, federal structure policy).^18,21^ However, despite legislation outlawing the caste system in Nepal since 1962, discrimination in accessing health services still continues due to a lack of state-run services, as well as denial and discrimination in the provision of health care to Dalits who seek health services.^22^ Typical discriminatory behaviors include refusing to enter Dalits’ houses or allowing them into your house, share food and water, seating places, transport, and generally refusing to touch. Health discrimination is likely to be seen mainly in areas where care is provided, which can be health centers or a patient’s own home.^23^ Research highlighted that Dalits are also more vulnerable to HIV (human immunodeficiency virus) partly due to high migration to escape from caste-based discrimination.^14^

While there are many papers written on caste, to date no systematic review has been conducted to explore the caste-based inequality in accessing health care services in South Asia. To bridge this gap, this review explores possible factors underlying discrimination and trends in discrimination and inequity over time. This review aims to addresses the research question, “What is the evidence that Dalits (ie, lower castes) have different health service utilization than higher castes in South Asia?” Our findings will be useful for policymakers and researchers alike.

## Methods

A systematic review was conducted in accordance with the PRISMA (Preferred Reporting Items for Systematic reviews and Meta-Analyses) as this allows inclusion of published papers to examine health discrimination on the basis of an individual’s caste.^24^ Population, exposure, and outcome framework^25^ was used to develop the research question and form the search strategy. This included the following: population—Dalits, untouchables, low caste, outcaste, minority group, socially excluded group, discriminated group, and exposure; discrimination—inequality, inequity, racism, barrier, violence, judged, prejudice, and outcomes; service uptake—motivation, hospital uses, health standard, health promotion, health equity, equality, health access, and health utilization.

The following databases were searched: CINAHL, Medline, SocINDEX, PubMed, Nepjol, JSTOR, ASSIA, and EBSCO Discovery Service for relevant articles. Table 1 shows different but interrelated search terms that varied depending upon the database. Boolean operator “OR” was applied in combining different search keywords for study population, exposure, and outcomes, whereas “AND” was applied to merge population, exposure, and outcomes. Proximity “N3” was applied to ensure the searched studies were health related. The parameters that framed the review included studies written in English and published in peer-reviewed journals.

**Table 1.** Search Strategy.

Database	Filter	Search terms	Results
My Search—Peer Reviewed	Language: English Date:19672019	(dalit* or untouchab* or “low caste*” or outcast* or minorit* or “social* exclu*” or “discriminated group*”) AND (discriminat* or inequal* or inequit* or racis* or barrier* or violen* or judg* or prejudice*) AND (((uptake* or motivat* or use* or adher* or promotion* or inequity or inequality) N3 health)) or “health access” or “health utilization” or “health utilization”	6444
CINAHL Complete—CINAHL Headings	Language: English Date:1981-2019	(Dalit* or Untouchab* or Low Caste* or Outcast* or Minorit* or Social* Exclu* or Discriminated Group*) AND Discriminat*or Inequal* or Inequit* or Racis* or Barrier* or Violen* or Judg* Or prejudice* AND (“Health Services Accessibility” or “Quality of Health Care” or “Attitude to Health” or “Attitude to Health Personnel” or “Patient Satisfaction” or “Health Knowledge” or “Health Status Disparities” or “Healthcare Disparities”)	2107
Medline Complete—MeSH Headings	Language: English Date:1950- 2019	(Dalit* or Untouchab* or Low Caste* or Outcast* or Minorit* or Social* Exclu* or Discriminated Group*) AND Discriminat*or Inequal* or Inequit* or Racis* or Barrier* or Violen* or Judg* Or prejudice* AND (“Health Knowledge, Attitudes, Practice” or “Quality of Health Care” or “Quality Assurance, Health Care” or “Health Care Quality, Access, and Evaluation” or “Health Services Accessibility” or “Attitude of Health Personnel” or “Attitude to Health” or “Patient Satisfaction” or “Patient Acceptance of Health Care” or “Health Status Disparities” or “Healthcare Disparities”)	6269
SocINDEX with Full Text—Subject Terms	Language: English Date:1987-2019	(Dalit* or Untouchab* or Low Caste* or Outcast* or Minorit* or Social* Exclu* or Discriminated Group*) AND Discriminat*or Inequal* or Inequit* or Racis* or Barrier* or Violen* or Judg* Or prejudice* AND (“Health of Minorities” or “Health Behavior–Research” or “Patient Satisfaction” or “Utilization of Health Facilities” or “Health Facilities” or “Health & Social Status”)	60
PubMed	Language: English Date: till 2019	(((Dalit* OR Untouchab* OR “Low Caste*” OR Outcast* OR Minorit* OR “Social* Exclu*” OR discriminated Group*)) AND health discrimination) AND (nepal OR india)	48
Nepjol	Language: English Date: till 2019	Dalit and health	30
JSTOR	Language: English Date: till 2019	(((dalits) AND (health)) AND (discrimination))	128
ASSIA	Language: English Date: till 2019	(Dalit* or Untouchab* or “Low Caste*” or Outcast* or Minorit* or “Social* Exclu*” or “Discriminated Group*”) AND (Discriminat*or Inequal* or Inequit* or Racis* or Barrier* or Violen* or Judg* Or prejudice*) AND ((uptake* or motivat* or uptake* and use* or Adher* or promotion*or inequity or inequality) N3 health)	20
Hand searches	Language: English Date: till 2019	Additional records through other sources	3
		Total	15 109

### Screening and Selection

**Figure 1.**
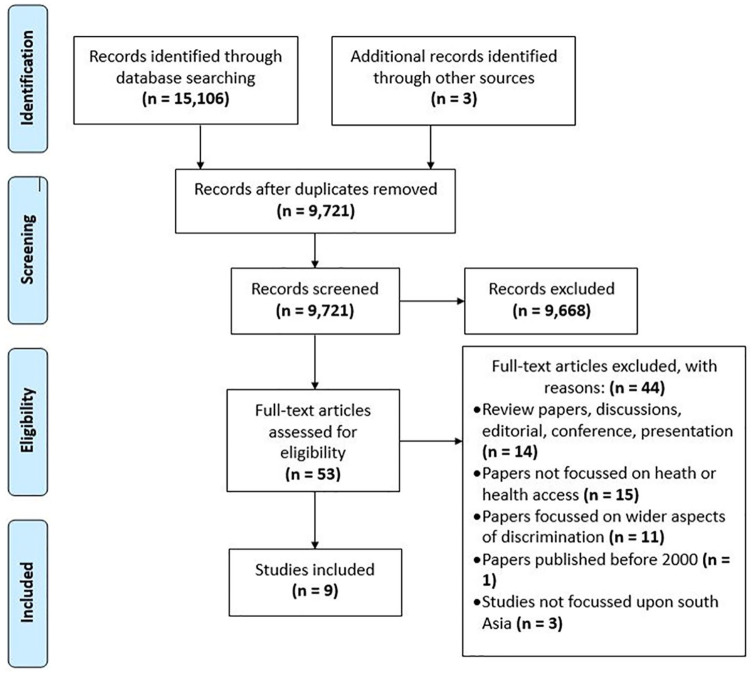
PRISMA flow diagram.^45^

Papers were selected using the 4-stage process suggested in PRISMA: identification, screening, eligibility, and included (Figure 1).

Records were identified through database searching (n = 15 106) and from other sources (n = 3), resulting in 15 109 records identified. After removing duplicates, 9721 articles were screened by reading the title and abstract against the inclusion/exclusion criteria. This included papers published between 2000 and 2019, primary research (qual/quant/mixed) secondary data analysis of routine data, focused on health, health access, health inequity, or discrimination against Dalit community, studies focused on south Asia, papers written in English and peer reviewed. At this stage, the majority of records (n = 9668) were excluded on the basis of main interest of paper not related to caste and health discrimination, language (not written in English), demographical location (research outside South-Asia), participants (population other than Dalits), and publications (not research paper). Ten percent of the rejected papers were also blind peer reviewed (EvT, VH, and PR) to ensure quality control.

In the third stage, all remaining 53 records full-text articles were reviewed by the first author against the inclusion/exclusion criteria and double-checked by the rest of the research team (EvT, VH, and PR) to reduce possible researcher bias. At this stage, 9 papers were selected and 44 papers were excluded largely due to study not being primary research, not focused on health or health access, focused on wider aspects of discrimination, not focused on South Asia, and published before 2000.

### Data Extraction

Data extraction was conducted by first author and reviewed by coauthors to ensure consistency. Any disagreement in the selection was resolved through discussion with other authors.

## Results

Nine studies that met inclusion criteria were included. Table 2 shows a summary of the appraised studies. Of the nine selected studies, two were qualitative studies, three quantitative, and four were mixed method. The selected South Asian studies were carried out between 2000 and 2019, mainly in India (n = 7) and Nepal (n = 2). These studies assessed caste-based discrimination in the health care sector.

**Table 2.** Summary Table.

Authors, year, and country	Main aims	Method and data collection/analysis	Participants	Results	Stated conclusion	Limitation, critical appraisal	Reviewer’s conclusion
Bhandari and Chan^30^ (2016), Nepal	Investigate caste/ethnicity-based inequity in women’s health service utilization, focusing on ANC in Nepal	Secondary cross sectional: Nepal Demographic Health Survey Data 2011, bivariate and multivariate analysis	4018 mothers aged 15 to 49 years who gave birth past 5 years	53% mother had ANC visit 4+ (mean 3.63, median 4.0), Hill Dalits and Terai Dalits were only 4%. Only 6% of disadvantages caste/ethnicity belongs to the wealthiest quintile.	Disadvantaged mothers using less ANC independently based on their caste/ethnicity and their household wealth. Advantaged mothers are also disadvantaged of utilizing ANC depending on wealth.	Secondary data, cross-sectional nature, not focus on quality visit. Appraisal: CASP, 2018 (75%, medium).	In-depth study association double discrimination: caste and wealth.
Kumar^31^ (2007), India	Explore the link between SHGs + women’s access to health services	Mixed method: survey, interviews, case studies, and focus group discussions	SHGs women (n = 200), family members, community leaders	84% SC used unlicensed “private doctors,” paid high charges. No change reported in health, health knowledge, health utilization, spending on food, basic needs compared with OBC. Participations’ health impact was reportedly greater for OBC women than SC.	Caste and class powerful in determining women’s access to health. Dependent on gender relations, income, education, and general standards of living. SHGs fail to overcome structural contexts hence failed to produce equitable health services to marginalized.	Small sample, number of participants other than SHGs women is not made clear. Appraisal: McGill, 2018 (85%, medium).	Women are discriminated on gender, caste, and class, information on double discrimination would be helpful.
Polit^35^ (2005), India	To show how the relative marginality of Dalits affects the well-being of Dalit women	Mixed method: Survey and ethnographic research	Garhwali (state) women central Himalayas of north India	First village: Dalits = poor, literate, high discrimination, and dependency. Second had 2 areas, occasional clashes, moderate dependency, employed, and educated people. Third village: only Dalits, less land, education, and jobs; no discrimination and dependency.	People’s affects well-being more than trans location. Dalit living exclusively Dalit village not consider themselves marginal and well-being = greater. Dalit in village with a high-caste majority will feel more marginal therefore, well-being likely to be less.	Small sample, limited information on methods, and data collection. Appraisal: McGill, 2018 (80%, medium).	No clear identified result. Dalits not marginal in all villages and level of discrimination, access to health and well-being are very different.
Priya and Sathyamala^36^ (2007), India	To explore level of ill health of people from low castes, capacities to respond to adult illness, and support needed	Cross-sectional Mixed methods: survey and interviews	1171 household Uttar Pradesh (UP) + 900 Tamil Nadu (TN) from SC, interviews = 62 in UP + 52 in TN	Two regions had distinctive health vulnerabilities and support systems. Death rates UP (9.4) and TN (11.4) not as expected. UP 19%/94% had treatment and in TN 97% with long-term illness had some treatment. Sources of treatment were loans. Stigma long-term illness not problem.	People who are poor and lower castes are not equally susceptible to HIV. Social cohesion provided security from impact of poor living and working conditions. Traditional forms of social cohesion are under stress and new forms are inadequate. No social norms protect women.	Methods and selection of participant not clearly explained. Appraisal: McGill, 2018 (85%, medium).	Discrimination on long-term illness is not a major issue; however, fear of stigma led to preventable death.
Mohindra et al^33^ (2006), India	Examine social patterning of women’s self-reported health status: Kerala; 2 hypotheses: (1) low caste and socioeconomic position is associated with worse health status, (2) associations between socioeconomic position and health vary across castes	Secondary cross-sectional data: household survey implemented by the Centre for Development Studies in 2003. Multilevel multinomial logistic regression model.	4196 non-elderly women of marital age (18-59 years).	Lower caste women, more likely never attended school and are predominantly wage laborers, OBC are slightly more likely to work as wage laborers and forward caste engage in nonwage activities. Odd rations poor perceived health and ADL.	Caste and socioeconomic interrelated; lower caste magnifies health inequity. Being both lower caste and poor can trap people into poor health than either inequality on its own. Implementing interventions that deal with caste and socioeconomic disparities to produce more equitable results than targeting either inequality in isolation.	Cross-sectional study, multilevel multinomial modes in ADL, self-perceived health, regular contact with professionals and attitudes and perceptions. Appraisal: CASP, 2018 (85%, medium).	Information on effects of gender inequality of women’s self-reported health status would be helpful.
George^38^ (2015), India	Examine Dalits in significant positions of rural health + improvement provisioning of health services in tribal India	Secondary analysis: National Sample Survey Office (un)employment (2011-2012)	National survey	Dalits are underrepresented in health professionals. Despite only 24% of rural population, other castes shares 40% in health professional. Shortages subcenters, PHCs, and CHCs.	Underrepresentation of Dalits in rural health care delivery due to untouchability. Indian health system is not equipped to address exclusion, which for urgent policy attention.	Secondary analysis of health work force. Appraisal: CASP, 2018 (80%, medium).	Further study on why in rural India significant jobs are likely to be taken by higher castes.
Daniel et al^37^ (2012), Nepal	Examine health care access to Dalits through experiences of stakeholders throughout health system	Ethnography, participatory approach: KI interviews and FGD, stakeholder, and institutional analysis	19 FGD and 19 KI totals (n = 209)	Dimension: info access, physical access, financial access, discrimination, and social capital restricting access to health services identified 5 themes: human rights education, health education, advocacy, public inclusion, and dialogue.	Dalits and non-Dalits less access to health services due to lack of resources, absence of monitoring health care, and problems decentralization also main causes of weak Nepali health system.	Less information: district due to language and geography—social relations for field visits. Appraisal: CASP, 2018 (70%, medium).	A table reflecting KI and FGD would be helpful in better understanding of results.
Verma and Acharya^34^ (2018), India	Explore health interaction of Dalit health staffs with non-Dalit care seekers and vice versa	Qualitative—in-depth interviews and 4 FGD, systematic random sampling; thematic analysis	20 ANMs, 20 ASHAs + 80 care seekers who delivered babies in last 6 months	5 Themes: Caste, perception and social identity, profession and social identity, maintaining identity, conflict and dilemmas, control and autonomy. Variation across caste of providers and seekers in shaping perception of each other.	Dalit providers lacked skills and health seekers are suspicious of their knowledge. Other staff limited interaction with Dalit care seekers and staff. Women faced gender and caste. Provider and seekers’ caste more weight than profession and need.	Small number, no justification how themes were identified, no written consent. Appraisal: CASP, 2018 (90%, high).	Clarification on how FGDs were conducted and further information on double discrimination would be helpful.
Rao^32^ (2015), India	Discuss key conceptual ideas of agency, voice, and interjectionally in relation to the role of marriage and sexuality in reinforcing caste and gender boundaries	Mixed method: Survey, In-depth interview, FGDs, and KI, narrative	Rural couples: 400 surveys, 40 in-depth interviews	Choice of marriage partner—arranged marriage among OBC, contestations among Dalits degree of control. Facing violence, resisting it, narrative by all women. Jobs are not easy for women, enhances dependence on their men.	More understanding needed on Dalit women’s “acceptance” of violence. Inseparability/lack of agency, of action/patience, as strategies to challenge hierarchy and strengthen their bargaining position.	Narrative research. Appraisal: McGill, 2018 (80%, medium).	More clear results, details related to data analysis, dowry-related violence/marriage.

Abbreviations: ANC, antenatal care; CASP, Critical Appraisal Skills Program; SHG, self-help groups; SC, scheduled castes; OBC, other backward classes; HIV, human immunodeficiency virus; ADL, activities in daily living; PHC, primary health center; CHC, community health center; FDG, focus group discussion; KI, key informant; ANM, auxiliary nurse-midwife; ASHA, accredited social health activist.

The selected nine studies were critically apprised using the CASP^26,27^ and McGill^28^ checklist, most scored average to moderate quality. From the nine studies, four themes were identified: stigma, poverty, culture and beliefs, and health care.

### Stigma

Stigma here refers to the stigma related to belonging to a particular group, in this case being a Dalit.^29^ Almost all studies identified issues related to caste-based stigma, however, four studies^30-33^ focused on the double discrimination of gender and caste experienced by Dalit women. Caste-based health discrimination was dominant across low-caste groups where basic health indicators for disadvantaged groups (including Dalits) were consistently poor in comparison with those in middle and upper castes.^30^ Caste is an important element in shaping individual’s social identity and their well-being.^34,35^

Dalits reported they were treated differently after people found out their caste status and those people then shared very little information about health services and programmes.^34^ Dalits live in an oppressive society, which impacts on every aspect of their life: they are not allowed to enter upper caste house, sit together, or even sit in the presence of upper caste people. People from low caste are also forbidden to offer food or water to upper caste people.^35^ Caste is one of the key factors of gender inequality, which is associated with poorer education, nutrition, and health as well as less access to human rights as illustrated by Dalit women being more vulnerable to diseases (malnutrition and anemia) and maternal mortality.^31^ The caste system is further gendered in terms of employment, including daily wages and long working days that makes it difficult for women to look after themselves in pregnancy and care for their offspring after birth which, in turn increases their dependence on men.^32^

Health outcomes of Dalit women are dependent on two major variables: caste and household wealth. Violence against women resulting from stress associated with unreliable work, low wage, and men being unable to perform their role as “provider.”^32^ Two studies identified domestic violence as a common issue with Dalit communities.^32,36^ Rao^31^ identifies that both alcohol consumption and violence are signs of men failing to control their jobs and kids, and then blaming women for their inability to perform as housewife. Dalit women are not allowed to travel alone; their mobility is restricted after marriage and they have little involvement in important household decisions including decision-related top-seeking health care, which can be one reasons why Dalit mothers have low levels of health service usage.^30^

Dalit women often suffer harassment from men including their husbands or have abusive relationships.^36^ When it comes to sexual intimacy, relationships between upper caste women and low-caste men is strictly prohibited, it is believed that this will pollute upper caste women, whereas sexual relations between upper caste men and low-caste women is not prohibited.^35^

### Poverty

Four studies^31,35-37^ identified financial limitations as a major barrier in accessing health care and in health-seeking behaviors. Dalits are generally poor and often not aware of free health services and government-provided health incentive schemes.^37^ Dalits are minority communities who have significantly less landholdings, have a lower socioeconomic status, and low literacy. They either work in upper caste people’s fields or in low-income jobs such as basket weaving.^35^ In contrast, upper caste groups have a higher health knowledge, better health access as well as better employment, and physical home environment compared with disadvantages caste.^31^ Bhandari and Chan^30^ found in their research that women from poor households are disadvantaged in terms of utilizing health services and most of Dalits live under the poverty line.

The connection between caste and occupation is complex. Lower caste women, despite nonengagement in education (as most never attended school), are more likely to be engaged in paid employment compared with women from other castes in society, as poorer women need to work to support their household.^33^ Education changes social interaction and job opportunities, which negatively affects Dalits as nearly 70% are illiterate, much lower than other lower castes.^32^ Conversely, the same study also identified that improvement in level of education among Dalits fueled violence due to refusal to work as submissive agricultural laborers.^31^

Kumar^30^ stated that private doctors who are usually unlicensed “quacks” exploit women due to their lack of education, charging high fees that results in Dalit women taking loans for treatment, perpetuating their poverty.^30^ He further added that without change in caste and class barrier, providing better health resources and improved health results are not possible, especially for women.^31^ Illness expenditures are mostly met by family members, loans from wider family, or self-help groups or banks.^36^ Two studies^31,36^ identified that taking out loans to cover health-related cost is common within Dalits communities and this further constrains their financial ability. Polit^35^ explained the hopelessness, depressed environment of Dalit society where individuals cannot afford hospital treatment or time to heal shows the extreme level of poverty in Dalits communities.^35^

### Cultures and Beliefs

Here any cultural factors or beliefs associated with health equity among Dalit communities are included. In South Asia, classifications based on caste and ethnicity are a main feature of social inequity, which is closely connected with Hindu beliefs.^30^ The cultural practices of discrimination affects one’s mental and social well-being.^35^ Despite regulations and laws prohibiting caste discrimination, continuous caste-based inequity creates hopeless and helpless situations for Dalits, which contributed to the development of alcoholism and self-harming behaviors. In response to this, some Dalits convert to Christianity to escape the caste system altogether.^36^ The caste system also influences marriages, as Dalits are less likely to be able to marriage a partner from a higher caste as marriages are expensive as large dowries are expected by higher caste families.^32^

In Polit’s study, people’s health and well-being were strongly connected to several deities, demons, and ghosts that live in their surroundings. Access to cures, ritual healing is restricted for Dalits due to the complex relationship between poverty and discrimination in society.^35^ Inequity leads to frustration, anxiety, and insecurities, which resulted in incidents of sprit possession by both male and females to express resulting unexplained death, while seeking exorcism treatment by the *ojha.*^36^

According to two studies, it is believed that caste people are not highly educated and cannot understand health information provided to them.^34,37^ They are ill-mannered and do not communicate properly due to lack of understanding and knowledge.^34^ Due to their limited access to care, Dalits lack knowledge about diseases and/or information about possible cures, which in turn leads to failing to identify symptoms or causes of illness.^37^

### Health Care

All nine studies identified that caste affects health issues, four focused^30-33^ on health issues related to women, one^36^ focused on health issues related to human immunodeficiency virus (HIV) and the remainder focused on general health and well-being issues related to Dalits. Kumar’s^31^ study asserts that disadvantaged women have a higher chance of severe illness and without any improvement in caste and class barriers, improved health resources and outcomes are almost impossible.^31^ In Uttar Pradesh, India, only 19% of people with long-term illness were treated due to poor availability of health services, high fees, and untrained providers.^36^ Poor health is connected with lower education level and having less land in low-caste women.^33^ Dalit women usually do not visit hospital for treatment due to the travel distance to the hospital and they cannot afford travel expenses or high treatment fees.^35^

Kumar indicated that in Bihar, India’s poorest state, local public medical services, often the only services affordable for Dalits, only addressed basic health needs.^31^ Despite developments in the Indian health structure including availability of subcenters, public health centers, and community health centers, poorer communities still experience a shortage of health institutions and skilled health workers.^38^ Similarly, in Nepal, the health care structure is weak and access is limited for both Dalits and non-Dalits. From skilled health workers to medicine, supplies, and other equipment are in short supply; therefore, patients are referred to higher level of care adding high fees and transportation costs with no guaranteed satisfactory care. Due to literacy issues, Dalits who travelled for better health care need support in filling in complex paperwork and they often struggle to get free access to services that are supposed to be free of charge.^37^

As previously mentioned, caste influences employment opportunities and few Dalits have gained health care positions such as general medical practitioners, specialist doctors, trained nurses, technicians, and associated health staff. Their low representation compared with other groups of society promotes a favorable environment for caste inequality.^38^ Dalit health workers including auxiliary nurse midwives suffered several difficulties; they were not treating well, patients and colleagues fail to follow their health advice and start taking them for granted due to their lower caste status.^34^

## Discussion

This review investigated caste-based inequity in health care utilization in South Asia and included nine selected studies. These research studies were carried out in different cities, counties with different study participants; however, most of them agreed there exists a connection between socioeconomic differences and health disparities.^30-38^ It was found that low socioeconomic status and holding less land is associated with poor health outcomes. Due to Dalits’ low status in Nepal and India, they have lower access to education and skilled well-paid employment results in lower household incomes.^39^ Dalits have lower occupational mobility, less land, poorer education, and worse jobs. Discrimination in occupation, prolonged poverty, and social stigma reduces their opportunity to access labor market in equal terms with non-Dalits; they also fail to get into occupations that did not conform to their low social and political status.^40^ Although law and policies have been introduced, socioeconomic hierarchies based on caste persist in South Asia.^41^

Dalit women are doubly disadvantaged due to their low-caste status as well as the lower status of women in Hindu society. Lower caste women have increased burdens and risks on their everyday life, including domestic violence and are more likely to experience severe sickness and limited treatment beyond locality. Self-help groups have helped caste women reduce reliance on moneylenders in the event of illness, to cover expensive private health services by providing limited credits.^31^ However, it is only available locally and not changing people’s attitudes toward discrimination. Caste/ethnicity contributes to women’s health, for example, higher caste groups comparatively derive better benefits from an antenatal care (ANC) program. Deprived caste/ethnicity group are disadvantages in terms of using ANC services compared with other caste groups. Similarly, mothers from wealthy household utilized ANC services more compared with poor households. However, when comparing caste intersecting household wealth results were slightly different. Disadvantaged women with lowest household wealth significantly used less ANC services compared with higher wealth with same household, similarly advantaged groups with lowest household wealth also significantly used ANC less, whereas mothers from both groups with better wealth used were significantly more likely to use ANC services. This shows the independent contribution of caste and wealth on health and contrary to the common beliefs that disadvantages groups are always disadvantaged.^30^

Dalit communities are not only affected by clinical issues but also a variety of sociocultural determinants; therefore, to improve their health and well-being better policies are needed as well as a willingness to tackle sociocultural determinants of health inequality with the government playing a central role.^40^ Caste influences are not limited to locational periphery and travels from village to the cities to all markets where “cultural and social relations play out”^42^ and affected the process of developments and educations. This review identified that the cultural practices of discrimination create psychological tensions disturbing mental health. Among Dalits mental health issues is often mistaken as being possessed by ghost. This kind of belief limits their access and understanding to better health services.^35^ Programs like SGH or ANC may not be enough to overcome caste and health attitudes and such programs may leave disadvantaged people behind in terms of health improvements. Therefore, the global policy agenda and national health system improvements need to focus on improving health inequalities across disadvantaged populations.

Caste and discrimination is largely invisible in discussions of Sustainable Developments Goals (SDGs).^43^ The SDGs of no poverty, good health and well-being, quality education, gender equality, and specially goal 10, reduced inequality for all, irrespective of age, sex, disability, race, ethnicity, origin, religion, economic, or other status will not be able to achieve without dealing with caste discrimination.^2^ Polit described how well-being in the three groups of Dalit villagers was affected by the circumstances of relative marginality as well as by general socioeconomic indicators. Dalits living together with a high-caste majority compared with Dalits living exclusively in a Dalit village are more marginal and have lower state of well-being.^35^ There is close interrelation between poor health, socioeconomic position, and education. Education is an exclusive measure of socioeconomic and socioeconomic position controls health behaviors.^33^

Health equity is also influenced by social status and perceptions of care providers and seekers, limiting their interaction with each other.^34^ Throughout history, Dalits have been classified as serving class and their only skill required is being able to serve.^44^ Non-Dalit health workers hold good understanding and respected in their community, whereas Dalits had limited mobility and nonacceptance within societies.^34^

## Conclusion

This review presents caste discrimination and health exclusion in South Asia and highlights the promotion of health and well-being of disadvantages castes as well as for the need for further study in other cultural contexts within South Asia. Research on Dalits often reports domestic violence, risk presence in everyday life, poor education, employment, health hierarchies, and inequities caused due to interconnection of caste, class, and gender. Class and caste inequities have become more severe in affective and determining opportunities to access to health care. Inequity in health can be visible on both sides in terms of care provider as well as seekers. This review highlights that due to poverty Dalits’ health seeking behavior is limited as they survive on daily wages and could not afford to lose their daily earnings. Similarly, deprived from accessing better health due to not being able to pay for expensive health services. Poverty also has an impact on education and health knowledge (ie, health literacy).

This review also shows the interrelation of caste and socioeconomic standard as a source of inequality, that is, the combination of being from lower caste and having low socioeconomic position results in poor health rather than just being poor. Dalit women face double discrimination due to their identity as women as well as low caste. Women’s interactions with education, income, and standard of living is limited, which leads them and their health very much dependent on existing gender relations. It will not be possible to boost the health of poor and Dalit women without decentralization and increasing local accessibility of health services. Dalits women’s problems are in addition to general weaknesses in health systems making accessing health care difficult for many people, not only for Dalits.

The evidence in this review indicates to the need for policy innovation and systematic and regular orientation program to address caste exclusion, remove barriers, and to provide support to Dalits development as well as pointing to the need of inequity discussions in global policy debate like Sustainable Development Goals.
